# Aspiration of the Perineum as a Means of Draining the Lower Part of the Pelvic Cavity

**Published:** 1877-12

**Authors:** 


Aspiration of the Perineum as a Means of Draining the Lower Part of the Pelvic Cavity.—Noticing the various procedures of meeting this difficulty, and the particular interest given it by the profession, I hope that a suggestion to this end may not be out of place. Among the various modes of draining the pelvic cavity, there seems to be none, as yet, that gives perfect satisfaction, none that affords entire safety to the patient, nor perfect evacuation of the pent-up fluid.

I think that a safe way of getting rid of the putrescent matter forming in this cavity, would be by aspiration through the perineum. As there would be no danger of puncturing the peritoneum, and having the advantage of not being troubled with fecal matter, or secretions of the womb, as in case of making openings through the rectal or vaginal walls, and as the perineum is at the bottom of this cavity, forming its floor, we would have no gravity to contend with. Whenever it is necessary to give vent to fluids forming in the pelvis, it seems to me that aspiration through the perineum at various intervals, would be less irritating than the continued presence of drainage tubes. Undoubtedly, the safest place to evacuate the pelvic cavity is through the perineum.—*John M. White, M.D., in Medical and Surgical Reporter.*

